# Antibiotic use in a tertiary healthcare facility in Ghana: a point prevalence survey

**DOI:** 10.1186/s13756-018-0299-z

**Published:** 2018-01-26

**Authors:** Appiah-Korang Labi, Noah Obeng-Nkrumah, Edmund Tetteh Nartey, Stephanie Bjerrum, Nii Armah Adu-Aryee, Yaw Adjei Ofori-Adjei, Alfred E. Yawson, Mercy J. Newman

**Affiliations:** 1 Department of Microbiology, Korle-Bu Teaching Hospital, P.O. Box 77, Accra, Ghana; 2 Department of Medical Laboratory Sciences, School of Biomedical and Allied Health Sciences, P.O. Box KB 143, Accra, Ghana; 3 Centre for Tropical Clinical Pharmacology and Therapeutics, School of Medicine and Dentistry, P.O. Box 4236, Accra, Ghana; 4 Department of Infectious Diseases, Copenhagen University Hospital, Rigshospitalet, Blegdamsvej 9, 2100 Copenhagen, Denmark; 5 Department of Surgery, University of Ghana School of Medicine and Dentistry, P.O. Box 4326, Accra, Ghana; 6 Department of Medicine, Korle-Bu Teaching Hospital, P.O. Box 77, Accra, Ghana; 7 Department of Community Health, School of Public Health, College of Health Sciences, University of Ghana, Accra, Ghana; 8 Department of Medical Microbiology, School of Biomedical and Allied Sciences, P.O. Box KB 143, Accra, Ghana

**Keywords:** Antibiotic, Ghana, Africa, Point prevalence, Antibiotic stewardship

## Abstract

**Background:**

The global rise and spread of antibiotic resistance is limiting the usefulness of antibiotics in the prevention and treatment of infectious diseases. The use of antibiotic stewardship programs guided by local data on prescribing practices is a useful strategy to control and reduce antibiotic resistance. Our objective in this study was to determine the prevalence and indications for use of antibiotics at the Korle-Bu Teaching Hospital Accra, Ghana.

**Methods:**

An antibiotic point prevalence survey was conducted among inpatients of the Korle-Bu Teaching Hospital between February and March 2016. Folders and treatment charts of patients on admission at participating departments were reviewed for antibiotics administered or scheduled to be administered on the day of the survey. Data on indication for use were also collected. Prevalence of antibiotic use was determined by dividing the number of inpatients on antibiotics at the time of survey by the total number of patients on admission.

**Results:**

Of the 677 inpatients surveyed, 348 (51.4%, 95% CI, 47.6–55.2) were on treatment with antibiotics. Prevalence was highest among Paediatric surgery where 20/22 patients (90.9%, 95% CI, 70.8–98.9) were administered antibiotics and lowest among Obstetrics patients with 77/214 (36%, 95% CI, 29.5–42.8). The indications for antibiotic use were 245/611 (40.1%) for community-acquired infections, 205/611 (33.6%) for surgical prophylaxis, 129/611 (21.1%) for healthcare associated infections and 33/611 (5.4%) for medical prophylaxis. The top five antibiotics prescribed in the hospital were metronidazole 107 (17.5%), amoxicillin-clavulinic acid 82 (13.4%), ceftriaxone 17(12.1%), cefuroxime 61 (10.0%), and cloxacillin 52 (8.5%) respectively. Prevalence of meropenem and vancomycin use was 12(2%) and 1 (.2%) respectively. The majority of patients 181 (52%) were being treated with two antibiotics.

**Conclusion:**

This study indicated a high prevalence of antibiotic use among inpatients at the Korle-Bu Teaching Hospital. Metronidazole was the most commonly used antibiotic; mainly for surgical prophylaxis. There is the need to further explore factors contributing to the high prevalence of antibiotic use and develop strategies for appropriate antibiotic use in the hospital.

**Electronic supplementary material:**

The online version of this article (10.1186/s13756-018-0299-z) contains supplementary material, which is available to authorized users.

## Background

The discovery of antibiotics in the twentieth century immensely changed medical practice. It allowed for treatment of life threatening conditions and the conduct of complex medical procedures with a reduced risk of infections. Over time, global overuse of antibiotics has emerged as a major problem [1, 2], with up to 50% of patients reported to receive unnecessary antibiotics [3].

Excessive use of antibiotics leads to the development of complications such as antibiotic related diarrhoea and healthcare associated infections [4, 5]. It is also a significant contributor to the development and spread of multidrug resistant bacteria, currently regarded as global public health crisis [6–9]. Antibiotic overuse is driven by prescribing habits of practitioners, which are dynamic and likely to change over time. These habits are affected by multiple factors including pathogen related factors such as changing resistance profiles [6–9], prescriber related factors [10, 11] and external factors such as pressure from the pharmaceutical industry [10–12]. It has been suggested that antibiotic consumption will increase with rising incomes in developing countries and better access to medical insurance [6, 13]. Thus efforts to promote rational antibiotic use and infection control in these regions are paramount [7]. Accurate information on the use of antibiotics are crucial to address the problem of antibiotic overuse and resistance [12]. In Africa, there are few published studies on antibiotic use among inpatients [14–16]. At the Korle-Bu Teaching Hospital (KBTH), Accra Ghana, a survey conducted in 2000 showed a 53% prevalence of antibiotic use among inpatients with metronidazole being the most commonly used antibiotic [17]. Unpublished data from a point prevalence study carried out in the Medical department of the same institution in 2012 showed that 67.9% of inpatients had been prescribed antibiotics.

Continuous evaluation of antibiotic use is important to preserve the effectiveness of antibiotics and minimize patient harm [13]. The WHO recommends surveillance of antibiotic use as a strategy for improving antibiotic use among patients and also for controlling antibiotic resistance [6]. Repeated point prevalence surveys of antibiotic use have been shown as a useful and cost effective way of evaluating antibiotic use in hospital [18, 19]. This study reports the results from a point prevalence survey of systemic antibiotic use conducted in 2016 among inpatients at the KBTH. The aim was to determine the prevalence of antibiotic use and indications for their use.

## Methods

### Study setting and design

The Korle-Bu Teaching Hospital is a 2000-bed tertiary referral hospital situated in Accra, Ghana with about 200 admissions per day [15]. The hospital covers all medical specialties and provides referral healthcare services to an estimated population of 24 million Ghanaians. The hospital has an estimated average bed occupancy rate of 66.1% (i.e., *n* = 1321 patients per 2000 beds) [20]. Acute care services provided by the hospital KBTH include internal medicine, general surgery, neurosurgery, orthopaedics, plastic surgery, opthalmology, ear, nose and throat, obstetrics and gynaecology, neonatal and adult intensive care, paediatrics, chest unit and cardiothoracic surgery. The point prevalence study involved the survey of inpatients records from hospital folders and was conducted in selected units of KBTH between February and March 2016. The survey instrument used in the study was adapted with modifications from the European survey of antimicrobial resistance [21]. Only folders of inpatients on admission before 8 am on the day of the survey were included in the study. The departments included in this study were General Surgery, Orthopaedics, Paediatric Surgery, Genitourinary, Neurosurgery, Child Health (except the neonatal intensive care unit where rehabilitation works were ongoing), Medicine, Obstetrics and Gynaecology. These departments have the highest number of patient admissions per day. Wards with patients not matching the inclusion criteria and those with only day cases were excluded. As per study protocol, we excluded the Plastic Surgey & Burns Unit where patients are routinely administered long-term antibiotics on admissions.

### Data collection

A multidisciplinary team of doctors and an infectious disease specialists conducted the survey at selected units. Training and piloting of the study instrument was conducted for the survey team prior to the start of the study. Briefly, the training session chaired by the lead investigator introduced survey personnel to the objectives of the study; the purpose of each item on the data collection tool including definition of terms and indicator codes; methods for assessment of individual patient data; and the roles and responsibilities of each survey personnel. The session was concluded with a 1-day pilot point-prevalence survey in a Medical ward. This session was conducted a week prior to study inception to allow for corrective action. The point prevalence survey was conducted from 8 am to 8 pm daily within a 2-week period. Data collection from each unit was completed within the 12-h period. The survey team performed retrospective data collection using standardized case report forms which primarily comprise a patient-level structured template for documenting antimicrobial use on the day of survey (Additional file 1). They reviewed patients’ folders and treatment charts and collected information on antibiotic use only for the survey date. Folders of patients undergoing same day treatment or surgery were excluded. Relevant data elements such as age, sex, ward, and total number of patients on admission on survey day were retrieved. Other information collected included antibiotics administered and route of administration, their dosages, dosing intervals and number of missed doses. In addition, patients’ clinical diagnosis and indications for antibiotic use (hospital- or community-acquired) were recorded. In every case, the survey team decided on clinical grounds whether the patient was infected or not according to guideline definitions [21]. Briefly, an active infection on the survey day was defined by the presence of signs and symptoms. Patients were considered infected even when signs and symptoms were no longer present but the patient was still receiving treatment for that infection on the date of the survey. The signs and symptoms were also reviewed to ascertain the indication for treatment (hospital-acquired, community-acquired, surgical prophylaxis and medical prophylaxis). The team referred to medical and nursing records and other relevant charts to determine whether the infection is a healthcare related. Clinical diagnosis of infections 48 h after admission was described as hospital-acquired infections. Infections occurring within 48 h of admission were categorized as community-acquired. The WHO anatomical therapeutic classification (ATC) of medicines was used for classifying drugs [22].

### Data handling and statistical analysis

Data was entered into MS Access® and exported into statistical package for social sciences (SPSS version 21) for cleaning and analysis. The prevalence of antibiotic use was defined as a percentage of the total number of patients on any systemic antibiotic at the time of survey against the number of patients on admission. Descriptive statistics (e.g., cross-tabulations, frequencies, and proportions) were used to examine data on antibiotic use. Proportions were compared using Chi-square tests.

## Results

A total 677 in-patient folders were reviewed from participating units/departments. The majority of included patients were admitted at the Obstetrics unit (*n* = 214), Child Health (*n* = 111), Medicine (106), Orthopaedics (*n* = 84), General Surgery (*n* = 55), Gynaecology (*n* = 38), Neurosurgery (*n* = 23), Urology (*n* = 24) and Paediatric Surgery (*n* = 22). The median age of included patients was 39 years and 54.2% were females. Adults (age > 15 years) comprised 71%, followed by children (29 days to 15 years old; 19.9%) and neonates (0–28 days; 9.1%).

### Prevalence and type of antibiotic use

**Fig. 1:**
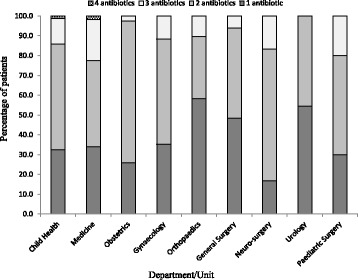
Number of antibiotics used per patient in the surveyed departments/units of Korle-Bu Teaching Hospital. A significant majority of patients on antibiotics received > 1 antibiotic regimen

**Fig. 2:**
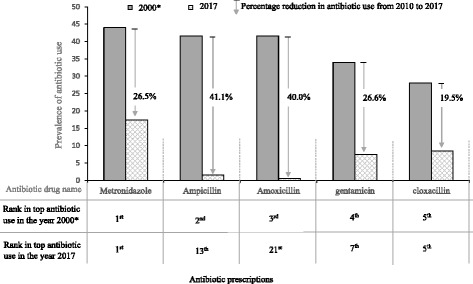
Top five antibiotic use in 2000 and 2017 at KBTH. Figures on antibiotic use in 2000 based on data by Newman, 2009 [17]

Table 1 shows the prevalence of antibiotic use in the participating hospital. In all, 348 (51.4%, 95% CI, 47.6–55.2%) of admitted patients received one or more antibiotics at the time of survey. Parenteral formulations constituted the significant majority of all antibiotic prescriptions (59.9%, *n* = 366/611). They were administered in 32.8% (*n* = 222/677) of admitted patients. Significantly fewer patients (18.8%, *n* = 127/677) received oral antibiotics. Across hospital units, the proportion of patients on antibiotics ranged from 36% (95% CI, 29.5–42.8) in Obstetrics to 90.9% (95% CI, 70.8–98.9) in Paediatric surgery. Of 348 patients on antibiotics, 127 (36.0%, 95% CI, 31.5–41.8) were on one antibiotic, 181 (52.0%, 95% CI, 46.6–57.4) were on two antibiotics, 38 (10.9%, 95% CI, 7.9–14.8) were on three antibiotics whilst 2 (0.6%, 95% CI, 0.1–2.3) were on 4 antibiotics (Fig. 1). The median number of antibiotics per patient was 2. A total of 611 antibiotic prescriptions were recorded. The top five percentage use by drug classes (ATC level 5) were as follows: penicillin based drugs (24.9%, *n* = 152), nitroimidazoles (17.5%, *n* = 107), 3^rd^ generation cephalosporins (13.8%, *n* = 84), 2^nd^ generation cephalosporins (10.0%, *n* = 61), and aminoglycosides (8.8%, *n* = 54). The five most commonly used generic antibiotics (Table 2) were metronidazole (17.5%, n = 107), amoxicillin-clavulanic acid (13.4%, *n* = 83), ceftriaxone (12.1%, *n* = 74), cefuroxime (10.0%, n = 61), and cloxacillin (8.5%, *n* = 50). The obstetrics unit accounted for the highest use of metronidazole (50.5%, n = 54) and amoxicillin-clavulanic acid (58.5%, *n* = 48) respectively. Figure 2 compares the top five antibiotic prescriptions at KBTH in 2000 [17] and 2017. In 2000, the most common antimicrobial in use at KBTH was metronidazole 212(44%), followed by ampicillin/amoxicillin 199(41.6%), gentamicin 168(34%) and cloxacillin 135(28%). In 2017, only metronidazole and cloxacillin remain in the top five antibiotics prescribed, albeit with significantly reduced percentage use. Cloxacillin recorded the least reduction (19.5%) in percentage antibiotic use, followed by metronidazole (26.5%), gentamicin (26.6%), amoxicillin (40.0%) and ampicillin (41.1%).

**Table 1:** Prevalence rates of antibiotic use across departments/units

Department	Patients on antibiotics		Antibiotic prescriptions			
	Number	% prevalence [95% CI]	Parenteral	Oral	Total	% prevalence [95% CI]
Obstetrics (n = 214)	77	36.0 [29.5-42.8]	46 (33.8)	90 (66.2)	136	16.5[13.8-19.5]
Child Health (n = 111)	77	69.4 [59.9-77.8]	120 (85.1)	21(14.9)	141	22.5[19.5-25.9]
Medicine (*n* = 106)	53	50.0 [40.1-59.9]	65 (64.4)	36 (35.6)	101	16.5[13.8-19.5]
Orthopaedics (n = 84)	48	57.1 [45.9-67.9]	26 (35.6)	47 (64..4)	73	13.3[10.4-16.2
General Surgery (n = 55)	33	56.9 [43.2-69.8]	28 (58.9)	24 (46.2)	52	8.6[8.7-11.4]
Paediatric Surgery (n = 22)	20	90.9 [70.8-98.9]	32 (84.2)	6 (15.8)	38	5.8[4.2-8.6]
Gynaecology (n = 38)	17	44.7 [28.6-61.7]	17 (53.7)	13(43.3)	30	4.5[3.1-6.4]
Neuro-surgery (n = 23)	12	52.2 [30.6-73.2]	21 (87.5)	3 (12.5)	24	3.6[2.4-53.8]
Urology (n = 24)	11	45.8 [29.8-74.3]	11 (68.8)	5 (31.2)	16	2.3[1.4-3.8]
All departments (n-677)	348	51.4 [47.6-55.2]	366 (59.9)^b^	245(40.1)^a^	611	

Prevalence was determined by dividing the number of inpatients on antibiotics at the time of survey by the total of patients on admission; *CI*, confidence interval; b > a at *p* < 0.05

**Table 2:** Proportion of prescribed antibiotics (ATC level 5) by Departments/units

Drug name (generic)		Department/Unit								
	Total	Child Health	Medicine	Obstetrics	Gynaecology	Orthopaedics	General Surgery	Neuro-surgery	Urology	Paediatric Surgery
		n, %	n, %^1^	n, %	n, %	n, %	n, %	n, %^1^	n, %^1^	n, %^1^
Metronidazole	107 (17.5)	4 (2.8)	13 (12.9)	54 (39.7)	9 (30.0)	3(4.1)	9 (17.3)	4 (16.7)	2 (12.5)	9 (23.7)
Amoxicillin- clavulanic acid	82 (13.4)	1 (0.7)	7 (6.9)	48 (35.3)	8 (26.7)	1 (1.4)	12 (23.1)	2 (8.3)	1 (6.3)	2 (5.3)
Ceftriaxone	74 (12.1)	20 (14.2)	27 (26.7)	4 (2.9)	4 (13.3)	1 (1.4)	2 (3.9)	7 (29.2)	1 (6.3)	8 (21.1)
Cefuroxime	61 (10.0)	6 (4.3)	2 (2.0)	13 (9.6)	5 (16.7)	28 (38.4)	5 (9.6)	1 (4.2)	–	1 (2.6)
Cloxacillin	52 (8.5)	28 (19.9)	10 (9.9)	1 (0.7)	–	5 (6.9)	–	6 (25.0)	–	2 (5.3)
Clindamycin	49 (8.0)	10 (7.1)	3 (2.9)	4 (2.9)	1 (3.3)	25 (30.1)	7 (13.5)	1 (4.2)	1 (6.3)	–
Gentamicin	46 (7.5)	30 (21.3)	3 (2.9)	2 (1.5)	1 (3.3)	1 (1.4)	2 (3.9)	1 (4.2)	–	6 (15.8)
Ciprofloxacillin	39 (6.4)	6 (4.3)	7 (6.9)	–	1 (3.3)	9 (12.3)	8 (15.4)	1 (4.2)	5 (31.3)	2 (5.3)
Azithromycin	21 (3.4)	–	13 (12.9)	5 (3.9)	1 (3.3)	1 (1.4)	–	–	1 (6.3)	–
Co-trimoxazole	14 (2.3)	7 (5.0)	7 (6.9)	–	–	–	–	–	–	
Meropenem	12 (2.0)	2 (1.4)	1 (1.0)	–	–	–	2 (3.9)	–	4 (25.0)	3 (7.9)
Amikacin	8 (1.3)	7(5.0)	–	–	–	–	–	Q	-a	–
Ampicillin	10 (1.6)	7 (5.0)	–	–	–	–	–	–	–	3 (7.9)
Crystal penicillin	7 (1.2)	6 (4.3)	1 (1.0)	–	–	–	–	–	–	–
Cefotaxime	6 (1.0)	6 (4.3)	–	–	–	–	–	–	–	–
Levofloxacin	5 (0.8)	–	3 (3.0)	–	–	1 (1.4)	1 (1.9)	–	–	–
Erythromycin	5 (0.8)	–	1 (1.0)	4 (2.9)	–	–	–	–	–	–
Clarithromycin	3 (0.5)	–	–	–	–	–	3 (5.7)	–	–	–
Nitrofuratoin	2 (0.3)	–	–	–	–	1 (1.4)	–	–	1 (6.3)	–
Ceftazidine	2 (0.3)	–	1 (1.0)	–	–	–	–	–	–	1 (2.6)
Amoxicillin	1 (0.2)	–	–	–	–	–	1 (1.9)	–	–	–
Cefixime	1 (0.2)	–	–	1 (0.7)	–	–	–	–	–	–
Doxycycline	1 (0.2)	–	1 (1.0)	–	–	–	–	–	–	–
Cefpodoxime	1 (0.2)	–	–	–	–	–	–	–	–	1 (2.6)
Nalidixic acid	1 (0.2)	1 (0.7)	–	–	–	–	–	–	–	–
Vancomycin	1 (0.2)	–	1 (1.0)	–	–	–	–	–	–	–
Total	611	141	101	136	30	73	52	24	16	38

*ATC,* Anatomic therapeutic classification; *n*=number; %, prevalence

### Indications for antibiotic use

Overall, 245 (40.1%) of 611 prescriptions were administered for community-acquired infections, 128 (21.0%) for hospital-acquired infections, 205 (33.6%) for surgical prophylaxis and 33 (5.4%) for medical prophylaxis (Table 3). The most prescribed antibiotic for surgical prophylaxis (*n* = 205) was metronidazole (32.2%, *n* = 66), followed by amoxicillin-clavulanic acid (25.9%, *n* = 53) and cefuroxime (13.7%, *n* = 28). For community-acquired infections, the three commonly prescribed antibiotics were ceftriaxone (18.0%, *n* = 44), cloxacillin and gentamicin (10.6%, *n* = 26). Of the 128 antibiotics used for hospital-acquired infections, the top three drugs were ceftriaxone (12.5%, 16.0), metronidazole (12.5%, 16.0), and cloxacillin (11.7%, *n* = 15). The proportion of antibiotic use by anatomic site is presented in Table 4. About a sixth (16.0%, *n* = 101) of all antibiotic prescriptions were for undefined reasons. The majority of antibiotics were prescribed for genitourinary and obstetric systems (25.2%, *n* = 154), and skin, soft tissue, bones and joints (19.4%, *n* = 119). The three most prescribed antibiotics for the former were metronidazole (40.3%, *n* = 62/154), amoxicillin- clavulanic acid (37.0%, *n* = 57/154), and cefuroxime (5.2%, *n* = 8/154). Clindamycin (30.3%, *n* = 36/119), cefuroxime (22.6%, *n* = 27/119) and ciprofloxacin (10.9%, *n* = 13/119) were the most indicated antibiotics for skin, soft tissue, bones and joints.

**Table 3:** Proportion of prescribed antibiotics (ATC level 5) by indication

Drug name (generic)	Total, %	Community acquired	Hospital acquired	Surgical prophylaxis	Medical prophylaxis
		n, %	n, %	n, %	n, %
Metronidazole	107 (17.5)	22 (9.0)	16 (12.5)	66 (32.2)	3 (9.1)
Amoxicillin- clavulanic acid	82 (13.4)	18 (7.4)	9 (7.0)	53 (25.9)	2 (13.4)
Ceftriaxone	74 (12.1)	44 (18.0)	16 (12.5)	13 (6.3)	1 (3.0)
Cefuroxime	61 (10.0)	24 (9.8)	7 (5.5)	28 (13.7)	2 (6.1)
Cloxacillin	52 (8.5)	26 (10.6)	15 (11.7)	8 (3.9)	3 (9.1)
Clindamycin	49 (8.0)	21 (8.6)	13 (10.2)	15 (7.3	–
Gentamicin	46 (7.5)	26 (10.6)	13 (10.2)	5 (2.4)	2 (6.1)
Ciprofloxaciin	39 (6.4)	17 (6.9)	14 (10.9)	8 (3.9)	–
Azithromycin	21 (3.4)	16 (6.5)	3 (2.3)	2 (1.0)	–
Co-trimoxazole	14 (2.3)	3 (1.2)	1 (0.8)	–	10 (30.3)
Meropenem	12 (2.0)	2 (0.8)	9 (7.0)	1 (0.5)	–
Amikacin	8 (1.3)	3 (1.2)	3 (2.3)	–	2 (6.1)
Ampicillin	10 (1.6)	7 (2.9)	–	2 (1.0)	1 (3.0)
Crystal penicillin	7 (1.2)	2 (0.8)	2 (1.6)	–	3 (9.1)
Cefotaxime	6 (1.0)	5 (2.0)	1 (0.8)	–	–
Levofloxacin	5 (0.8)	1 (0.4)	3 (2.3)	1 (0.5)	–
Erythromycin	5 (0.8)	2 (0.8)	–	–	3 (9.1)
Clarithromycin	3 (0.5)	2 (0.8)	–	1 (0.5)	–
Nitrofuratoin	2 (0.3)	–	1 (0.8)	1 (0.5)	–
Ceftazidine	2 (0.3)	–	2 (1.6)	–	–
Amoxicillin	1 (0.2)	1 (0.4)	–	–	–
Cefixime	1 (0.2)	–	–	1 (0.5)	–
Doxycycline	1 (0.2)	1 (0.4)	–	–	–
Cefpodoxime	1 (0.2)	–	1 (0.8)	–	–
Nalidixic acid	1 (0.2)	1 (0.4)	–	–	–
Vancomycin	1 (0.2)	1 (0.4)	–	–	–
Total	611(100)	245	128	205	33

*ATC,* Anatomic therapeutic classification, *n* number;* %*, prevalence

**Table 4:** Proportion of prescribed antibiotics (ATC level 5) by anatomic site

Drug name (generic)	Anatomic sites								
	Total	CNS	OTH	UT	GIT	SSTBJ	GUOB	RESP	Undefined
		n, %		n, %	n, %	n, %	n, %	n, %	n, %
Metronidazole	107	10 (9.3)	–	–	15 (14.0)	6 (5.6)	62 (57.9)	10 (9.3)	4 (3.7)
Amoxicillin- clavulanic acid	82	1 (1.2)	1 (1.2)	–	2 (2.4)	8 (9.8)	57 (69.5)	10 (12.2)	3 (3.7)
Ceftriaxone	74	16 (21.6)	2 (2.7)	1 (1.4)	9 (12.2)	4 (5.4)	8 (10.8)	24 (32.4)	10 (13.5)
Cefuroxime	61	1 (1.6)	–	4 (6.6)	5 (8.2)	27 (44.8)	9 (14.8)	11 (18.0)	4 (6.6)
Cloxacillin	52	13 (25.0)	1 (1.9)	–	2 (3.9)	13 (25.0)	–	–	23 (44.2)
Clindamycin	49	1 (2.0)	–	–	1 (2.0)	36 (73.5)	2 (4.1)	5 (10.2)	4 (8.2)
Gentamicin	46	3 (6.5)	2 (4.3)	1 (2.2)	3 (6.5)	6 (13.0)	1 (2.2)	5 (10.9)	25 (54.4)
Ciprofloxacin	39	1 (2.6)	–	5 (12.8)	9 (23.1)	13 (33.3)	6 (15.4)	2 (5.1)	3 (7.7)
Azithromycin	21	1 (4.8)	–	–	–	–	1 (4.8)	18 (85.7)	1 (4.8)
Co-trimoxazole	14	2 (14.3)	–	–	–	–	–	11 (78.6)	1 (7.1)
Meropenem	12	–	–	4 (33.3)	1 (8.3)	1 (8.3)	1 (8.3)	1 (8.3)	4 (33.3)
Amikacin	8	1 (12.5)	–	–	–	–	–	–	7 (87.5)
Ampicillin	10	–	–	–	2 (20.0)	–	–	3 (30.0)	5 (50.0)
Crystal penicillin	7	–	1 (14.3)	–	1 (14.3)	–	1 (14.3)	3 (42.9)	1 (14.3)
Cefotaxime	6	2 (33.3)	–	–	–	–	–	–	4 (66.7)
Levofloxacin	5	–	–	1 (25.0)	–	2 (40.0)	–	–	2 (40.0)
Erythromycin	5	–	–	–	–	1 (20.0)	4 (80.0)	–	–
Clarithromycin	3	–	–	–	3 (100)	–	–	–	–
Nitrofuratoin	2	–	–	1 (50.0)	–	1 (50.0)	–	–	–
Ceftazidine	2	–	–	1 (50.0)	–	–	–	1 (50.0)	–
Amoxicillin	1	–	–	–	1 (100)	–	–	–	–
Cefixime	1	–	–	–	–	–	1 (100)	–	–
Doxycycline	1	–	–	–	–	–	1 (100)	–	–
Cefpodoxime	1	–	–	–	–	1 (100)	–	–	–
Nalidixic acid	1	–	–	1 (100)	–	–	–	–	–
Vancomycin	1	1 (100)	–	–	–	–	–	–	–
Total	611	52	7	19	54	119	154	104	101

*CNS* central nervous system; *OTH*, others; *UT* urinary tract, *GIT* gastrointestinal tract, *SSTBJ* skin, soft tissue, bone and joints, *GUOB* genitourinary and obstetrics, *RESP* respiratory; *n*, number;* %*, prevalence

## Discussion

We conducted a point prevalence survey of antibiotic use among inpatients of the KBTH in Ghana. The study identified a high prevalence of antibiotic use with nearly 51% of inpatients receiving antibiotics. The antibiotics were mainly used for treatment of community-acquired infections (40.1%) and surgical prophylaxis (33.6%). The prevalence level reported in this study is comparable to a prevalence rate of 53% recorded in a previous study among inpatients at KBTH in 2000 [17]. These rates may represent a relatively stable antibiotic use prevalence over the period.

The prevalence rate is however lower than 59.9% prevalence of antibiotic use recorded among out patients in primary healthcare facilities in Ghana [23]. It is also lower than 64.6 and 67.4%, which are the prevalence rates recorded in hospitals in Benin and Vietnam, countries with similar developmental profiles as Ghana [14, 24]. The prevalence of antibiotic use in this study is however comparable to the prevalence of 49.9% [13] recorded in acute care hospitals in the United States of America; and higher than antibiotic prescribing rates in Europe, which range between 30.1–35% [21, 25]. Our indicated prevalence is also lower than that reported in the ARPEC study (36.7%) which surveyed children from 226 hospitals in 41 countries across six continents [16].

High rates of antibiotic use are usually associated with inappropriate use of antibiotics and the development of antibiotic resistance and healthcare associated infections [4, 8, 26]. In Vietnam and Turkey, antibiotics were deemed to be prescribed inappropriately in 30.8 and 46.7% of cases [12, 24]. High rates of antibiotic use observed at the KBTH may be due to lack of antibiotic formulary or uniform standard protocols for managing infections for the hospital, despite presence of a national standard treatment guidelines [27]. Although this study was not designed to evaluate the appropriateness of antibiotic use, it is expected that with high prevalence of antibiotic use, a significant proportion of the use in KBTH may be inappropriate or unnecessary. This finding thus presents an opportunity to reduce antibiotic consumption in the hospital.

The prevalence of antibiotic use was highest among paediatric surgical patients (90.6%) and lowest among obstetric patients (36.0%). This fact could be explained by the high use of prophylactic antibiotics among paediatric surgical patients. The majority of patients (52%) were on two antibiotics. This is comparable to findings from the survey conducted in 2000, where 42.6% of patients in the hospital were on two antibiotics [17]. A significant proportion of prescribed antibiotics (59.9%) were administered via the parenteral route. Highest use of parenteral antibiotics was found in the neurosurgical unit, child health and paediatric surgery unit. Similar rates especially among children has been reported in other studies [16]. Such high usage of parenteral antibiotics may be associated with unsafe needle use [28]. Although the prevalence of antibiotic use in the hospital seems to be stable, the agents used are changing. In the year 2000 the top five agents were metronidazole, followed by ampicillin/amoxicillin, gentamicin and cloxacillin [17]. In this study, 26 different antibiotics were used among inpatients with metronidazole, amoxicillin-clavulanic acid, ceftriaxone, cefuroxime and cloxacillin contributing to more than 50% of all antibiotics prescribed. These agents were mainly used for surgical prophylaxis and treatment of community-acquired infections. The apparent change in agents over the years may point to increasing reports of antibiotic resistance from Ghana over the past decades [29–31]. Use of carbapenems (2.0%) and glycopeptides (0.2%) in the hospital were recorded to be low. These low rates may reflect the unavailability of these agents on the national health insurance scheme essential drug list and their association with high out-of-pocket purchase cost. It is however important to maintain low use of such agents in the hospital in the long term to avoid development of carbapenem resistant *Enterobacteriaceae* and vancomycin resistant enterococcus. Both pathogens are associated with very poor clinical outcomes [8, 32].

The majority (40%) of antibiotics were prescribed for community-acquired infections. Community- acquired infections were commonly treated with ceftriaxone (18%), with infections of the respiratory tract being in the majority (32.4%). This reflects a common clinical practice of using ceftriaxone as a first line agent for community-acquired pneumonia. However, only moderate penicillin resistance has been reported among *Streptococcus pneumoniae* isolates in Ghana [33, 34]. Frequent use of ceftriaxone may be a contributory factor to the high prevalence of extended spectrum beta-lactamase (ESBL) producing organisms seen at the KBTH [35]. The most commonly used antibiotics were metronidazole and amoxicillin-clavulanic acid and the main indication was surgical prophylaxis. This was mainly accounted for by patients from the obstetrics and gynaecology department. Surgical prophylaxis in this group of patients is effective in reducing post-operative complications [36]. It is common practice in the hospital to give long term antibiotics to patients undergoing caesarean section as prophylaxis, although it has been found not to be beneficial [36]. High usage of anti-anaerobic antibiotics like metronidazole is associated with elimination of gut anaerobes leading to the growth promotion of nosocomial pathogens [37]. This may promote the development of hospital-acquired infections such as vancomycin resistant enterococcus infections [38]. This study supplements data on antibiotic use in the KBTH and Ghana. It sets a bench mark for which other studies may be compared with and highlights areas for possible improvement in antibiotic use if stewardship programmes are to be implemented.

Our study has some limitations. It is a one site study and results may not be extrapolated to other health facilities. The prevalence of antibiotic use may be under estimated since the survey was not conducted in every unit of the hospital. However, based on 62.5% [20] bed occupancy of the hospital and number of patients surveyed we believe our data is a good reflection on the current state of antibiotic use in the hospital.

## Conclusion

In this point prevalence survey, we found a high prevalence of antibiotic use among inpatients of the KBTH with a relatively high prevalence among paediatric patients. There is high use of metronidazole and amoxicillin-clavulanic acid and low percentage use of vancomycin and meropenem. Majority of the antibiotics were used treatment of community-acquired infections and surgical prophylaxis. Attempts aimed at reducing antibiotic use in the hospital should be focused on the use of the top five antimicrobial agents as well as antibiotics used for surgical prophylaxis. There is also the need to further explore the factors contributing to the high prevalence of antibiotic use and the changing epidemiology of antibiotic use in the hospital.

## Additional file

Additional file 1: Antibiotic use survey instrument. (PDF 439 kb)

## Abbreviations

ATC: Anatomical therapeutic classification
CI: Confidence interval
CNS: Central nervous system
GIT: Gastrointestinal tract
GUOB: Genitourinary and obstetrics
KBTH: Korle-Bu Teaching Hospital
RESP: Respiratory
SSTBJ: Skin, soft tissue, bone and joints
UTI: Urinary tract
WHO: World Health Organization
