# Supplementary material for: Antibiotic use in a tertiary healthcare facility in Ghana: a point prevalence survey
Source: Antimicrob Resist Infect Control. 2018 Jan 26;7:15. doi: 10.1186/s13756-018-0299-z (PMC5787245; doi:10.1186/s13756-018-0299-z)
Supplement: Additional file 1: — Antibiotic use survey instrument. (PDF 439 kb) [file 13756_2018_299_MOESM1_ESM.pdf]

Project: Antibiotic use in Korle-Bu Teaching Hospital

Antibiotic use survey instrument

Date of survey: **Date of admission** **Survey no:** **Department/Unit:**

Mode of payment: A.NHIS ☐ B. Self-funded ☐ C. Private insurance ☐ **Age:** **Sex:** M ☐ F ☐ **Duration of admission:**

Drug	Date started	Date stopped	Unit dose[1]	Doses per day[2]	Route[3]	Diagnosis[4]	Indication[5]	Immuno-suppression[6]	Foreign material[7]	Culture pre-therapy[8]	Reason in notes[9]	Assessment[10]	Missed doses in admission[11]

Survey instrument adapted with minor modificationsfrom the European Surveillance of Antimicrobial Consumption (ESAC) Point-Prevalence Survey of Antibacterial Use in 20 European Hospitals

Project Lead: Dr Appiah-Korang Labi
Contact: Department of Medical Microbiolog: Phone, 0244863056
